# A Randomised Double-Blind Placebo-Controlled Clinical Trial of Fish Oil (Omega-3) in Sjögren’s Syndrome Patients in Erbil-Iraq

**DOI:** 10.31138/mjr.090224.rdb

**Published:** 2025-01-21

**Authors:** Ziad Shafeeq Al-Rawi, Aryan Mohamadfatih Jalal, Ibtihal Hikmat Hameed

**Affiliations:** 1 Department of Rheumatology, College of Medicine University of Baghdad, Baghdad, Iraq; 2 Department of Rheumatology, Kurdistan Board for Medical Specialties, Erbil, Iraq; 3 Department of Rheumatology, Hawler Medical University, Erbil, Iraq

**Keywords:** Sjögren’s disorder, omega three fatty acid, dry mouth, dry eye

## Abstract

**Introduction:**

Sjögren’s syndrome (SS) is an autoimmune condition that primarily targets glands that are exocrine. Fish oil supplements have been explored for their potential to decrease pain, reduce stiffness in the morning, and improve joint tenderness among rheumatic disease patients.

**Aim of study:**

to assess effectiveness of omega-3 fatty acids for controlling symptoms of dry eye and dry mouth in individuals diagnosed with Sjögren’s syndrome.

**Patients and Methods:**

A randomised, double-blind, placebo-controlled clinical trial was conducted over a two-month period. clinical trial registration (ID: NCT05005806). Dry eye symptoms were assessed using a six-point scoring system (0–3). Dry mouth symptoms were evaluated using a visual analogue scale (VAS). Secondary outcomes included Schirmer’s test and sialometry test.

**Results:**

The analysis included a total of 104 people with Sjögren’s syndrome. The mean score of dry eye symptoms was significantly lower in omega-3 Group (4.85 ± 4.10 SD, 95% CI: 3.75, 5.95) compared to the placebo group (8.27 ± 5.72 SD, 95% CI: 6.60, 9.93; P value = 0.001). Schirmers test after treatment, improved significantly to normal values in both groups which was slightly better among the omega-3 group. Sialometry tests indicated normalisation of salivary flow rate in the omega-3 group (2.07 ± 1.67 SD, 95% CI: 1.63, 2.52) (P value = 0.053).

**Conclusion:**

Omega-3 fatty acids effectively improved dry mouth and dry eye symptoms. Furthermore, this led to significant normalisation of salivary flow rate. While Schirmer’s test results improved in both groups, the differences between the omega-3 and placebo groups were insignificant.

## INTRODUCTION

Sjögren’s syndrome (SS) stands as a prevalent auto-immune disease predominantly affecting the exocrine glands. Its clinical course typically unfolds as sicca symptoms and tiredness gradually progress, some individuals may also suffer other symptoms outside of the gland.^1^ The hallmark of SS lies in the pervasive dryness of mucosal surfaces, encompassing the mouth, eyes, skin, nose, pharynx, larynx, and vagina.^2^ Despite its broad impact, SS often eludes timely diagnosis, either due to the challenge physicians face in recognising its cardinal features or patients’ reluctance to seek medical attention for dry mouth and eyes.^3^

Dry eye syndrome (DES) constitutes a multifaceted ailment involving the tear film and ocular surface, reflecting as elevated tear film osmolality and inflammation of the ocular surface.^4–6^ Studies have explored the potential ameliorative effects of oral supplement with omega three polyunsaturated fatty acids (ω-3 PUFAs) on lacrimal function tests in DES patients.^7–9^

Xerostomia, or dry mouth, results from insufficient saliva production by the salivary glands, often stemming from medication side effects, aging, or radiation therapy for cancer.^10^ Omega-3 fatty acids including Eicosapentaenoic acid (EPA), docosahexaenoic acid (DHA), and alpha-linolenic acid (ALA), play vital roles in modulating metabolism of prostaglandin to promote the synthesis of anti-inflammatory prostaglandins.^11^ Inflammation a key player in DES, manifests through heightened cytokine concentrations in the tear film of dry eye cases.^12^

Given the anti-inflammatory potential of ω-3 PUFAs, their role in alleviating symptoms in Sjögren’s syndrome like dry eye and dry mouth becomes paramount.^13^ Thus, this double-blinded randomised clinical trial aims to ascertain the efficacy of omega-3 supplement in treating sicca symptoms associated with Sjögren’s syndrome.

## AIM OF THE STUDY

The goal of this study is to evaluate the efficacy of including omega-3 fatty acids into the diet as a means of managing symptoms and indicators of dry eye and dry mouth in individuals diagnosed with Sjögren’s syndrome.

## PATIENTS AND METHODS

A randomised, double-blind, placebo-controlled clinical trial was conducted at the Rheumatology Outpatient Clinic of Rizgary Teaching Hospital, Erbil, Iraq, spanning from September 2021 to February 2023. A total of 126 patients were assessed for eligibility, with 104 patients ultimately completing the study. Participants, care providers, and investigators were blinded to the types of medication administered to patients throughout the study, while only the pharmacist was aware of the medication allocation until study completion. Participants were allocated randomly into two groups employing web-based randomised programme http://www.randomizer.org). Group 1 was given omega-3 dietary supplements (omega-3 group) while Group 2 was given a placebo.

The capsules and packaging for both groups were identical in shape, colour, and weight (1000 mg). Each patient in both groups received two capsules daily for two months.

Endpoints were assessed at baseline upon initiation of treatment and at the second visit after two months. Clinical evaluations and laboratory investigations were conducted at each visit. All enrolled patients (including early <6 months and established > 6 months) received standard treatments throughout the study period, including hydroxychloroquine (200 mg twice daily) and NSAIDs as needed and tolerated.

### Questionnaire design

A structured questionnaire, serving as a case sheet, was meticulously crafted to gather comprehensive information regarding patients’ demographic data, Sjögren’s syndrome current signs and symptoms, and history of chronic diseases. The questionnaire included sections for assessing patients’ visual analogue scale (VAS) scores for dry eyes and dry mouth, as well as results from Schirmer’s test and sialometry test. These were conducted at baseline and during the second visit after two months.

### Inclusion Criteria

1. Age must be between 18 and 70 years old.
2. Having the ability to give informed consent.
3. The diagnosis of Sjögren’s syndrome is determined using the categorisation criteria released in 2016 by the American College of Rheumatology (ACR) and the European League Against Rheumatism (EULAR).

### Exclusion Criteria

1. Presence of any pre-existing ocular disease or current use of eye drops.
2. Use of medications including beta-blockers, diuretics, anticholinergics, antidepressants, antihista-mines, pilocarpine, or cevimeline.
3. History of diabetes or psychiatric disorders.
4. Pregnancy or lactation.
5. Diagnosis of malignancy.
6. Smoking habit.
7. Diagnosis of other connective tissue diseases.

### Primary outcome

1. The main outcome measures the change in subject symptoms of dry eye, encompassing six symptoms (burning, foreign body sensation, itchy, dryness, mucous discharge, photophobia), the scoring for each symptom ranges from 0 to 3 where 0 indicates the absence of the symptom, 1 indicates occasionally present, 2 indicates frequently present, and 3 indicates always present. The overall score extends from 0 to 18, classifying the degree of severity as mild (0–6), moderate (6–12) severe (12–18) on the visual analogue scale.
2. Dry mouth was assessed using the inventory xerostomia score, consisting of 11 questions rated on a visual analogue scale ranging from 1 to 5 indicating the frequency from never to very often.

### Secondary outcome

1. Schirmer’s tear test was conducted by instructing the patient to sit on an examining chair, placing a test strip with a round wick at the lower eyelid’s junction, and measuring the length of the moistened area after five minutes. A normal result was considered when the length was ≥5mm.
2. Sialometry test measured salivary flow, and unstimulated whole saliva was collected for the period of 15 minutes, the volume was recorded and considered to be normal flow, when it was equal or more than 1.5ml/15 min.

## ETHICAL CONSIDERATION

Consent was gathered from all patients in written form and ethical approval was received from the Ethics and Scientific Committees of the Medical Research Ethical Approval committee at the University of Baghdad College of Medicine (Approval No. 3, dated July 28, 2021). The study was also registered on clinicaltrials.gov in 5^th^ of August 2021 with registration ID number (NCT05005806).

## STATISTICAL ANALYSIS

Data was recorded on a specially designed questionnaire, entered SPSS version 25.0, and analysed using appropriate statistical methods. Continuous data was presented as 95% confidence intervals (CI), mean (SD), while categorical variables were presented as absolute numbers and percentages. The significance level was set at ≤ 0.05, and comparisons among patient groups were made using T-tests, Chi-square tests, or Fisher’s exact test when applicable. Results were presented in tables and figures.

## RESULTS

**Figure 1.**
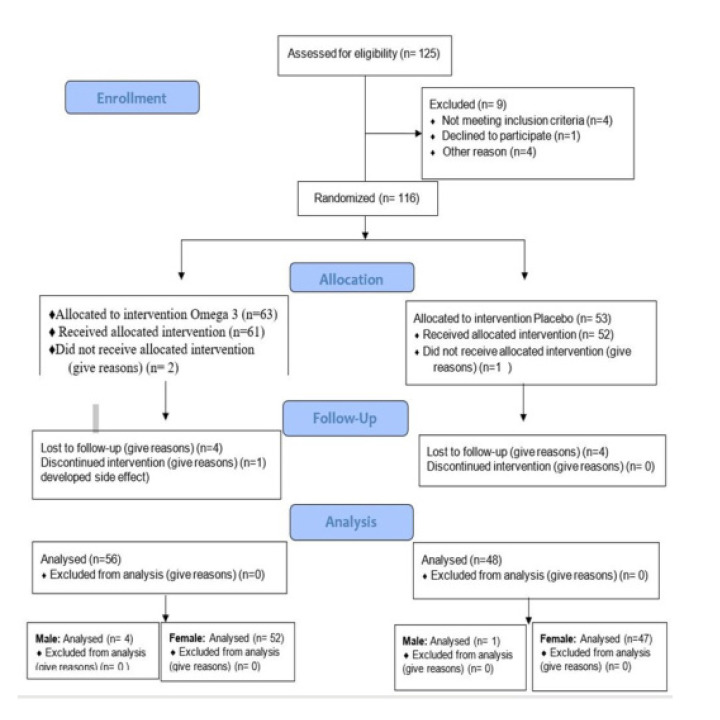
Consort 2010 flow diagram.

Out of 126 patients assessed for eligibility, 10 of them were excluded; 4 didn’t meet the inclusion criteria, 1 refused enrolment, and 5 because of poor compliance. 126 patients were randomised into two groups, 63 in the omega-3 arm and 53 in the placebo arm. Final analysis was conducted on only 104 patients who attended their last visit (56 in the omega-3 group, and 48 in placebo group) (**Figure 1**).

This study included a sample of 104 individuals diagnosed with Sjögren’s syndrome who were randomly and equally separated into two groups: the omega-3 group and the placebo group. The findings shown in **Table 1** indicate there is no differences between sociodemographic characteristics studied in both groups. As shown in **Table 2**, the omega-3 group Schirmer’s Test at baseline and at last visit were (2.38±2.13, 6.63±5.98) respectively, (p value was<0.001). There was a significant improvement in Schirmer’s test results post treatment in the omega-3 and placebo group. Schirmer test results at baseline and follow-up were (2.69±3.9, 5.58±6.68) respectively and the p value was 0.01. Improvement in Schirmer test results in the omega-3 group was better when compared to the placebo group, but the differences were insignificant.

**Table 1.** Sociodemographic features of 104 patients diagnosed with primary Sjögren Syndrome.

		**Omega-3 Group**	**Placebo Group**	

		**N. (%)**	**N. (%)**	**P value**
**Gender**	**Female**	52(92.9%)	47(97.9%)	
	**Male**	4(7.1%)	1(2.1%)	0.299
**Age**		53.48±12.49	52.64±11.33	0.723
**BMI**		31.22±6.04	31.37±6.47	0.542
	**Smoker**	3(5.4%)	4(8.3%)	0.744
**Smoker**	**Nonsmoker**	49(87.5%)	42(87.5%)	
	**Ex-smoker**	4(7.1%)	2(4.2%)	
**Duration of Sjögren**	**Early**	26(46.4%)	22(45.8%)	0.952
**Syndrome**	**Established**	30(53.6%)	26(54.2%)	
**Parotid Swelling**	**Yes**	54(96.4%)	42(87.5%)	0.091
	**No**	2(3.6%)	6(12.5%)	

BMI: body mass index; Early: <6 months; Established: >6 months.

**Table 2.** Schirmer’s test results in both groups at baseline and after treatment with omega-3 and placebo.

**Groups**		**Mean**	**Std. Deviation**	**95% CI of the Difference**		

				**LCI**	**UCI**	**P value**
**Omega-3**	Schirmer test at baseline	2.38	2.13	1.81	2.95	<0.001
	Schirmer test at last visit	6.63	5.98	5.03	8.23	

**Placebo**	Schirmer test	2.68	3.93	1.54	3.83	0.01
	at baseline					
	Schirmer test	5.58	6.67	3.64	7.52	
	at last visit					

As shown in **Table 3**, the stratification between subgroups showed that both groups (omega-3 and Placebo) had Schirmer test results that were near to each other at baseline and at last visit with insignificant p values.

**Table 3.** Stratification of both groups (early and established) in Schirmer test.

	**Group**	**Duration of disease**	**N**	**Mean**	**SD**	**P value**
**Omega-3**	**Schirmer test at baseline**	Early	26	2.8077	2.31981	
		Established	30	2.0167	1.92302	0.53

	**Schirmer test at last visit**	Early	26	7.2885	4.87067	
		Established	30	6.0667	6.83895	0.63

**Placebo**	**Schirmer test at baseline**	Early	22	2.1136	2.10403	
		Established	26	3.1731	4.98987	0.25

	**Schirmer test at last visit**	Early	22	5.7273	6.63455	
		Established	26	5.4615	6.84240	0.91

Early: <6 months; Established: >6 months.

The main symptoms that showed improvement with omega-3 were itching, mucous discharge and photophobia; with their p values being (0.02, <0.001, <0.001) respectively. The differences between the last visit and baseline in the above-mentioned symptoms were significant as shown in **Table 4**.

**Table 4.** Six dry eye symptoms at baseline and at last visit after two-month treatment period.

**Eye symptoms**	**Mean difference (95% CI)**	

		**P value**
Burning sensation	−0.40(−1.30,0.50)	0.468
**At baseline**		

Burning Sensation	−0.52(−0.94, −0.09)	0.200
**Last visit**		

Itching	−0.08(−0.51,0.33)	0.228
**At baseline**		

Itching	−0.65(−1.04, −0.24)	0.023
**Last visit**		

Foreign body sensation	−0.02(−0.45,0.41)	0.137
**At baseline**		

Foreign body sensation	−0.63(−1.04, −0.22)	0.064
**Last visit**		

Dryness	0.01(−0.28,0.32)	0.571
**At baseline**		

Dryness	−0.62(−1.06, −0.18)	0.339
**Last visit**		

Mucous discharge	0.06(−0.32, 0.45)	0.997
**At baseline**		

Mucous discharge	−0.42(−0.74, −0.10)	<0.001
**Last visit**		

Photophobia	0.16(−0.31, 0.65)	0.707
**At baseline**		

Photophobia	−0.55(−0.98, −0.12)	<0.001
**Last visit**		

**Figure 2.**
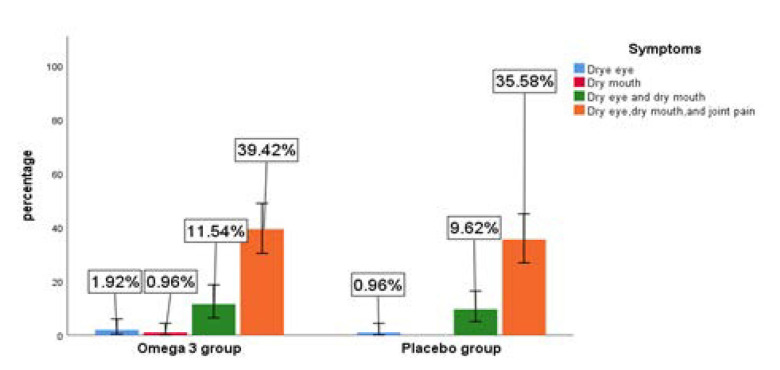
Symptoms of Sjögren’s syndrome in both groups.

As shown in **Figure 2**, the most common symptoms were a combination of dry eye, dry mouth, and joint pain amongst the omega-3 and placebo groups. **Table 5** shows that the Mean of Dry eye symptoms at baseline (10.44±4.08SD,.70 ± 4.90) in the omega-3 and placebo groups were close to each other, with a severe score rating. Post-intervention, those in the omega-3 group had a mean dry eye symptom score that was lower (4 .85 ± 4.10 SD, CI %95; 3.75,5.95), putting them in the mild to moderate camp when compared to the placebo group (8.27±5.72 SD, CI %95; 6.60,9.93), which were ranked moderate to severe (P value = 0.001).

**Table 5.** Mean of dry eye symptoms in both groups.

		**No.**	**Mean**	**SD**	**95% CI for Mean**		

					**LCI Bound**	**UCI Bound**	**P value**
**Dry eye symptoms at baseline**	Omega-3	56	10.44	4.08	9.35	11.54	
	Placebo	48	10.70	4.90	9.28	12.13	0.767
	Total	104	10.56	4.46	9.69	11.43	

**Dry eye symptoms at last visit**	Omega-3	56	4.85	4.10	3.75	5.95	
	Placebo	48	8.27	5.72	6.60	9.93	0.001
	Total	104	6.43	5.18	5.42	7.44	

Score: 0–6 mild, 6.1–12 moderate, 12.1–18 severe dry eye.

**Table 6** showed that the mean xerostomia inventory score of dry mouth symptoms at baseline (2.83±0.91,2.81 ± 0.74) in the omega-3 and placebo groups were close to each other. Post-intervention, those in the omega-3 group had a lower mean of dry mouth symptoms (1.91± 0.84 SD) versus the placebo group (2.35±1.006) (who scored moderate to severe) with a significant P value 0.017).

**Table 6.** Dry mouth symptoms by xerostomia brief inventory in both groups.

**Dry mouth symptoms**		**No.**	**Mean**	**SD**	**95% CI for Mean**				

					**LCI Bound**	**UCI Bound**	**Min**	**Max**	**P value**
**At baseline**	Omega-3	56	2.83	0.91	2.58	3.07	0.80	5.00	
	Placebo	48	2.81	0.74	2.59	3.02	1.70	4.70	0.961
	Total	104	2.82	0.83	2.66	2.98	0.80	5.00	

**At last visit**	Omega-3	56	1.91	0.84	1.69	2.14	0.50	5.00	
	Placebo	48	2.35	1.00	2.06	2.64	1.10	4.90	0.017
	Total	104	2.12	0.94	1.93	2.30	0.50	5.00	

**Table 7** results showed Sialometry test post intervention became normalised in the omega-3 group (2.07±1.67SD,95%CI;1.63,2.52) when compared to the placebo group (1.55 ± 0.83 SD, %CI;1.31,1.79) (with a significant P value).

**Table 7.** Sialometry saliva flow measure in both groups.

		**95% CI for Mean**									

		**No.**	**Mean**	**SD**	**LCI Bound**	**UCI Bound**	**Minimum**	**Maximum**	**P value**
**Sialometry at baseline**	Omega-3	56	0.99	0.62	0.83	1.16	0.00	3.00	0.989
	Placebo	48	1.00	0.68	0.80	1.19	0.00	3.50	
	Total	104	0.99	0.64	0.87	1.12	0.00	3.50	

**Sialometry at last visit**	Omega-3	56	2.07	1.67	1.63	2.52	0.00	12.00	0.053
	Placebo	48	1.55	0.83	1.31	1.79	0.10	3.50	
	Total	104	1.83	1.37	1.57	2.10	0.00	12.00	

## DISCUSSION

As there is no curative medication for treatment of Sjögren’s disorder, treatment is typically symptomatic and includes various therapies. The aim of therapy is to improve the quality of life by treating sicca symptoms, fatigue and by controlling other systemic manifestations of the syndrome. Pharmacological and non-pharmacological agents are used for this purpose. There are limited numbers of randomised controlled clinical trials published in this field and so any new drug that proves beneficial in managing this condition would be highly welcomed by physicians. There is evidence to support the fact that omega-3 fatty acids have anti-inflammatory properties.^**14**^

To test the hypothesis that omega-3 fatty acids could improve sicca symptoms, we conducted a randomised, double-masked, placebo-controlled trial involving 104 Sjögren’s syndrome patients. We found significant improvements when it came to dry mouth with normalisation of the sialometry test (during the last visit) amongst the omega-3 group. We also observed improvement significantly in dry eye symptoms and insignificant improvements of Schirmer test (when compared to placebo group patients). The findings of this research are equal with the findings of Papas A. et al., which indicated that therapy with omega supplementation could improve salivary flow and tendency was identified in the subjective feeling of oral and ocular involvement.^15^

Baseline assessment included examination of unstimulated salivary flow using sialometry, which showed a significant increase and normalisation during the final visit among patients receiving omega-3 when compared to the placebo group. In the research conducted by Singh et al. The study demonstrated that treatment with n-3 was not substantially better to wheat germ oil therapy. Yet, each group had a rise in unstimulated and stimulated salivary flow rate over three months, findings of this research support the idea that n-3 supplementation and maybe wheat germ oil have been successful in relieving the mouth dryness of individuals with Sjögren’s disease.^16^

Dry eye symptoms are a prevalent issue globally and can significantly impact individuals’ productivity. Common issues associated with dry eye include diminished visual acuity and reading challenges. and night driving challenges. In our study, we observed significant improvements in main eye symptoms post-treatment with omega-3, including itching, mucous discharge, and photophobia. These findings align with those of another study by another author despite them using a different formulation of omega-3 (GLA and n-3 PUFA) for a duration of 6 months (which also improved ocular irritation symptoms).^17^

In a double-blind study^18^ comparing omega-3 fatty acid to placebo, similar reductions in mean scoring of eye symptoms were observed among the omega-3 group compared to placebo group, which was consistent with our findings. However, they used a lower dose of omega-3 (500 mg BID) compared to our study (1000 mg BID). Additionally, Schirmer’s test values significantly improved among the omega-3 group in their study, whereas in ours, both groups demonstrated a substantial enhancement in Schirmer’s test measurements. It was noted (in our study) that a slightly better effect was observed in the omega-3 group, though the differences in improvement between the groups were insignificant, likely due to the consistent use of hydroxychloroquine tablet doses in both groups.

A meta-analysis and systematic review^19^ evaluating the efficacy of hydroxychloroquine (HCQ) in treating primary Sjögren syndrome found significant improvements in oral symptoms, unstimulated salivary flow rate (uSFR), the inflammatory indicators include ESR and C-reactive protein CRP plus immunoglobulin IgM levels. Still, the administration of HCQ did not lead to improvement in organ specific systemic complications in areas such as joints, lungs, nerves, lymphatic system, and kidneys. Contrastingly, a retrospective study reported sustained improvement in local symptoms such as ocular and mouth symptoms including discomfort and dryness as well as systematic manifestations including arthralgias and myalgias following 3 years of HCQ treatment.^20^

A systematic review and statistical analysis of randomised controlled trials investigating the effectiveness of polyunsaturated fatty acids (PUFAs) in treating dry eye syndrome, notable decreases in the ocular surface disease index providing evidence that PUFA supplementation could be a promising therapy for dry eye syndrome.^21^

Another multicentre, double-blind clinical trial^9^ provided evidence that found no significant difference in outcomes between patients with dry eye disease who received supplements that included 3000 mg of n–3 fatty acids for 12 months and those who assigned a placebo. This was inconsistent with our findings, there were variations in the qualifying criteria dosage of n−3 fatty acids, content of the placebo, length of supplementation, criteria for using alternative treatment for dry eye condition, dietary behaviours of the participants and outcome measures.

A different meta-analysis has presented data indicating that the intake of omega-3 fatty acids effectively improves both the symptoms and signs of dry eye in individuals diagnosed with dry eye.^22^

In another double-masked study, polyunsaturated fatty acids have been proven to be effective in alleviating symptoms related to dry eye disease, although the differences were insignificant. This discrepancy could be attributed to the intake of omega-6 alongside omega-3 in their study, which may have less impact on reducing the anti-inflammatory mechanism.^23^

## CONCLUSIONS

Omega-3 fatty acids effectively improved dry mouth and dry eye symptoms. Furthermore, they led to significant normalisation of salivary flow rate. While Schirmer’s test results improved in both groups, the differentiation between omega-3 and placebo groups were insignificant.

## CONFLICT OF INTEREST

Authors declare that they have no conflict of interest.
